# Exploring the biological role of postzygotic and germinal de novo mutations in ASD

**DOI:** 10.1038/s41598-020-79412-w

**Published:** 2021-01-11

**Authors:** A. Alonso-Gonzalez, M. Calaza, J. Amigo, J. González-Peñas, R. Martínez-Regueiro, M. Fernández-Prieto, M. Parellada, C. Arango, Cristina Rodriguez-Fontenla, A. Carracedo

**Affiliations:** 1 Grupo de Medicina Xenómica, Fundación Instituto de Investigación Sanitaria de Santiago de Compostela (FIDIS), Universidade de Santiago de Compostela, Santiago de Compostela, Spain; 2 Genomics and Bioinformatics Group, Center for Research in Molecular Medicine and Chronic Diseases (CiMUS), Universidade de Santiago de Compostela, Av Barcelona 31, 15706 Santiago de Compostela, Spain; 3 Fundación Pública Galega de Medicina Xenómica (FPGMX), Centro de Investigación Biomédica en Red, Enfermedades Raras (CIBERER), Universidad de Santiago de Compostela, Santiago de Compostela, Spain; 4 Centro De Investigación Biomédica en Red de Salud Mental (CIBERSAM), Hospital General Universitario Gregorio Marañón, Instituto de Investigación Sanitaria Gregorio Marañón, IiSGM, School of Medicine, Universidad Complutense, Madrid, Spain

**Keywords:** Genetics, Neuroscience

## Abstract

De novo mutations (DNMs), including germinal and postzygotic mutations (PZMs), are a strong source of causality for Autism Spectrum Disorder (ASD). However, the biological processes involved behind them remain unexplored. Our aim was to detect DNMs (germinal and PZMs) in a Spanish ASD cohort (360 trios) and to explore their role across different biological hierarchies (gene, biological pathway, cell and brain areas) using bioinformatic approaches. For the majority of the analysis, a combined ASD cohort (N = 2171 trios) was created using previously published data by the Autism Sequencing Consortium (ASC). New plausible candidate genes for ASD such as *FMR1* and *NFIA* were found. In addition, genes harboring PZMs were significantly enriched for miR-137 targets in comparison with germinal DNMs that were enriched in GO terms related to synaptic transmission. The expression pattern of genes with PZMs was restricted to early mid-fetal cortex. In contrast, the analysis of genes with germinal DNMs revealed a spatio-temporal window from early to mid-fetal development stages, with expression in the amygdala, cerebellum, cortex and striatum. These results provide evidence of the pathogenic role of PZMs and suggest the existence of distinct mechanisms between PZMs and germinal DNMs that are influencing ASD risk.

## Background

Autism Spectrum Disorder (ASD) is a neurodevelopmental disorder (NDD) characterized by deficits in communication and social interaction together with restricted interests and repetitive behaviors^1^. ASD prevalence among children in the United States stands at around 1.5% and has rapidly risen in recent years. In addition to the core symptoms of ASD, other conditions such as epilepsy or intellectual disability are often present. Comorbidity is a characteristic of ASD that can appear at any time during child’s development. Since many ASD cases with comorbidity have a clear genetic background and early detection is key for intervention, the genetic diagnosis in this type of cases is a challenge^2^.

Twin and family studies have estimated ASD heritability to be about 80% and subsequent genetic studies have demonstrated that the largest part of this heritability (50%) is explained by common variation^3,4^. However, de novo rare genetic variation (minor allele frequency < 0.1%), including small insertions and deletions (indels), copy number variants and single nucleotide variants confers higher individual risk^5–7^. Germinal de novo mutations (DNMs) occur within germ cells and they are transmitted to the offspring when the zygote is formed after fertilization. Thus, every single cell line of the resulting embryo will carry an identical genetic load. Another type of DNMs, postzygotic mutations (PZMs), arise during zygote mitosis, leading to a mosaic of genetically different cell lines^8^. The frequency of mutagenesis and the generation of PZMs is increased prior to gastrulation and neurogenesis^9^.

PZMs involved in ASD pathogenesis are usually detectable through deep sequencing of brain tissues. However, this technique often entails a huge challenge due to the inability to obtain ASD brain samples^10^. In contrast, next generation sequencing technologies can be used to detect mosaic mutations in peripheral blood of affected individuals by increasing the depth of coverage^11^. Thus, it is possible to obtain enough sequencing reads containing the reference and the alternate allele to accurately calculate the alternate allele frequency (AAF)^12^. In PZMs, the AAF value shifts from the expected 50/50 ratio for heterozygous germinal mutations. High coverage whole exome sequencing (WES) (depth > 200×) provides enough sensitivity to detect PZMs presenting AAF values as lower as 15%^13,14^. It is worthy to note that most WES studies have missed PZMs due to the commonly employed pipelines. The development of new variant calling pipelines is therefore needed and some efforts have been done in this regard^15–18^.

It has been estimated that 7.5% of DNMs are PZMs that contribute about 4% to the overall architecture of ASD. PZMs have been identified in high-confidence ASD risk genes. Other novel ASD candidate genes such as *KLF16* and *MSANTD2,* were discovered after studying the contribution of PZMs to ASD risk in large collections of ASD probands^18^. This points to the fact that some genes carry a larger number of mutations in a mosaic state than other genes. In addition, a detailed analysis of non-synonymous PZMs has revealed that these variants are mainly found in brain-expressed genes and in Loss-of-function (LoF)-constrained exons. The spatio-temporal analysis across different developmental stages also points to brain areas, like the amygdala, that have not been previously highlighted by other WES studies in which PZMs were not considered^16,18^.

The relevance of PZMs in the pathogenesis of ASD and the biological processes in which genes carrying PZMs are involved, remain largely unexplored. Moreover, the contribution of PZMs to the phenotypic presentation is another subject that should be studied in more detail using large-scale studies. Hence, it is suspected that ASD probands carrying mosaic mutations might be less affected than probands carrying germinal mutations as it happens in other NDDs such as Proteus syndrome or several brain malformations^19,20^. Therefore, the main aim of this study was to accurately detect DNMs (germinal and PZMs) in a cohort of Spanish trios with ASD (360). The novel DNMs detected in the Spanish cohort were combined with a list of DNMs previously published by the Autism Sequencing Consortium (ASC)^18^ in a cohort of 5947 families (4032 ASD trios and 1918 quads) in order to study if different ASD risk genes tend to accumulate one or another type of mutations using different bioinformatic approaches. In addition, the different biological implications of germinal and PZMs in ASD were explored through enrichment analysis approaches, which have not been applied before to this class of mutations across different hierarchical levels (gene, GO terms, neuronal cell types and brain areas) (Additional file 4. Fig. S1).

## Methods

### Subjects

DNA was extracted from peripheral blood of the Spanish ASD samples (360 trios; unaffected parents and affected proband) using the GentraPuregene blood kit (Qiagen Inc., Valencia, CA, USA). Subjects from Santiago (N = 136) were recruited from Complexo Hospitalario Universitario de Santiago de Compostela and Galician ASD organizations. Subjects from Madrid (N = 224) were recruited as part of AMITEA program at the Child and Adolescent Department of Psychiatry, Hospital General Universitario Gregorio Marañón. Only individuals 3 years old or older were included. All participants had a clinical diagnosis of ASD made by trained pediatric neurologists or psychiatrists based on the Diagnostic and Statistical Manual of Mental Disorders, Fourth Edition Text Revision and Fifth Edition (DSM-IV-TR and DSM-5) criteria. The Autism Diagnostic Observation Schedule (ADOS) and the Autism Diagnostic Interview-Revised (ADI-R) were also administered when necessary. Informed consent signed by each participating subject or legal guardian and approval from the corresponding Research Ethics Committee were obtained before the start of the study. All participants, parents or legal representatives provided written informed consent at enrollment and the study was conducted according to the declaration of Helsinki.

### Sample quality control and DNMs detection

#### Data processing and annotation

WES of DNA extracted from the Spanish 360 trios was performed by the ASC (https://genome.emory.edu/ASC/)^21^. One multi-sample VCF with the raw results was retrieved from the ASC. Individual files containing coding variants per individual were obtained using bcftools and were annotated using SnpEff (Genomic variant annotations and functional effect prediction toolbox) version 4.3 T (http://snpeff.sourceforge.net/).

#### Sample specific quality control

To check family relationships in the Spanish cohort (360 trios), information of Mendelian error counts was obtained using the "–mendel” option available in VCFtools (http://vcftools.sourceforge.net/). Samples whose Mendelian errors significantly deviated from the expectation were not considered for subsequent analysis.

To identify discrepancy between nominal designed and genetically determined sex the “–sexcheck” option in PLINK was used to infer correct sex from genotypes on chromosome X and Y.

Finally, to identify outlier samples in the Spanish cohort (360 trios), the “pseq i-stats” command in PLINK was used. Samples in which any of the following parameters: count of alternate, minor, heterozygous genotypes, number of called variants or genotyping rate deviated more than 4 SD from the mean were eliminated. Therefore, the whole trio was dropped if any member was considered an outlier.

The samples of the Spanish cohort (360 trios) that passed all the quality controls mentioned above were the same as those included in Satterstrom et al.^21^.

#### DNMs detection

To detect DNMs in the Spanish cohort (360 trios), defined as those mutations that are strictly present in probands and not in parents, the filtering options published by Lim et al. were employed^18^. In this study, variants classified as PZMs were resequenced by three different sequencing technologies reaching a high validation rate (87–97%).

Briefly, we define DNMs as those variants whose genotypes were 1/0 or 1/1 in probands and 0/0 in parents. Then, variants with GQ ≥ 20 and alternate read depth ≥ 7 were considered. Variants that present two or more alleles in the ExAC database (http://exac.broadinstitute.org/) were filtered out. Inframe indels were also filtered and only biallelic DNMs were considered. In addition, we filtered out variants that were less than 20 base pairs apart from each other to reduce false positives, and variants whose RVIS (Residual Variation Intolerance Score) retrieved from ExAC was higher than 75% were also filtered out. RVIS is designed to rank genes in terms of whether they have more or less common functional genetic variation relative to the genome-wide expectation given the amount of apparently neutral variation the gene has. Intolerant genes are more likely to be better candidates in NDDs. Thus, RVIS values represented as percentiles reflect the relative rank of the genes, with those genes above 75th percentile being the most tolerant and therefore less likely to harbor mutations with a role in ASD.

SnpEff was employed to classify exonic variants according to the definition of their predictive impact: high, moderate and low impact on the canonical transcript. Low impact variants included silent mutations, moderate impact variants included missense mutations and high impact included splicing and nonsense mutations. Only base substitutions were considered so frameshift variants were filtered out.

Two different in silico prediction tools (CADD and SIFT) were used to classify missense mutations. Probably damaging mutations were those predicted as damaging by SIFT and variants with CADD score > 20 (Additional file 1; Table S1).

Finally, DNMs were classified as germinal or PZMs based on the AAF (number of alternate reads/(total number of reference + alternate reads)). DNMs with an AAF ≥ 0.40 were classified as germinal and DNMs with an AAF < 0.40 were classified as PZMs^18^.

90 samples from the Spanish cohort (360 trios) were already analyzed by Lim et al.^18^ and they were used as positive controls to check if the detection of PZMs in the Spanish cohort was accurately made. Therefore, it was proved that most of DNMs were accurately detected and classified as germinal or PZMs (Additional file 1; Tables S1, S2).

For the majority of the analysis, we used a dataset called “combined cohort” (N = 2171) that includes the non-synonymous DNMs detected in the 360 Spanish trios plus the non-synonymous DNMs identified in individuals with ASD sequenced by the ASC and published previously. Duplicated variants in both cohorts were eliminated (Supplementary Table 3 of Lim et al.)^18^ (Additional file 1; Table S1 and S3). For some analysis, we also defined a control cohort of healthy siblings published by the ASC (same sequencing depth and variant calling procedures than the probands of the Spanish cohort) (N = 288)^1,18^ (Additional file 1; Table S4).

### Transmission and de novo association test (TADA-denovo)

TADA-Denovo (http://www.compgen.pitt.edu/TADA/TADA_guide.html#tada-analysis-of-de-novo-data-tada-denovo) was run to discover and to prioritize ASD risk genes for both DNMs (germinal and PZMs) in the Spanish cohort (N = 360) (Additional file 1; Table S1) and in the combined dataset (N = 2171) (Additional file 1; Table S3). TADA takes into account the mutational burden of the genes as well as the multiple mutational classes^22^. TADA was independently run in two different gene-sets for the Spanish cohort (genes harboring PZMs (PZMs genes) in the Spanish cohort = 105; genes harboring germinal DNMs (Germinal genes) in the Spanish cohort = 181) and for the combined cohort (PZMs genes in the combined cohort = 362; germinal genes in the combined cohort = 1210) (Additional file 2; Tables S5, S6, S7 and S8). The control cohort (N = 288) was employed to set up and to estimate the parameters needed by TADA^18^ (Additional file 1; Table S4).

Two classes of DNMs were included in the analysis: LoF and probably damaging missense mutations. To set up mutational rates for each mutational category, we used the per gene mutation rates table data computed by Samocha et al.^23^ and then, the following formula was applied to calibrate them: LoF; (nonsense + splice) x (syn_obs_/syn_exp_) and probably damaging missense; missense × (N_Prob.damaging_ /N_allmissense_) x (syn_obs_/syn_exp_). Syn_obs_ is the observed number of synonymous mutations in the control cohort of unaffected siblings (N = 119)^18^, and syn_exp_ is the expected number of synonymous DNMs in the same cohort calculated from the sum of per-gene synonymous DNMs rates (2*n*µ) (N = 79.04). N_prob.damaging_ is the number of probably damaging missense mutations in the control cohort (N = 212) and N _allmissense_ were the total of missense mutations in the control cohort (N = 296). To estimate the relative risk (γ) for each mutational category, we calculated the burden (ƛ) of mutations of each type in cases (Spanish cohort) over controls (LoF = 2.21; probably damaging missense = 1.36). Then we applied the following formula to calculate relative risk: ɣ = 1 + (ƛ − 1)/*π,* where *π,* the fraction of risk genes, was set as 0.05 (the default parameter). Finally, by running TADA-Denovo with the parameters described above, uncorrected *p* values for each gene were calculated obtaining null distributions (N repetitions = 10,000). TADA-Denovo computes BF (Bayesian Factor) to each gene. To determine an appropriate threshold that allows declaring a “significant gene”, TADA uses the Bayesian FDR approach to control for the rate of false discoveries. q-values for each gene were calculated using the Bayesian FDR approach provided by TADA. Manhattan plots which show the results of TADA *p* values (− log10) for the combined cohort (PZMs and germinal mutations) were done with R package qqman^24^.

Genes with FDR < 0.1 (germinal genes) and FDR < 0.3 (PZMs genes) were classified according to SFARI criteria (https://gene.sfari.org/database/gene-scoring/). Moreover, OMIM database (Online Mendelian Inheritance in Man) (https://www.omim.org/) was consulted to search for Mendelian diseases related to these genes.

### Gene-set enrichment analysis of PZMs and germinal mutations

Gene-set enrichment analyses of those genes carrying missense and nonsense DNMs (germinal and PZMs) was done by DNENRICH^25^. DNENRICH estimates the enrichment of DNMs within pre-defined groups of genes accounting for gene size, tri-nucleotide context and functional effect of the mutations. DNMs included in this analysis were germinal and PZMs identified in the Spanish cohort (germinal DNMs = 236; PZMs = 164) (Additional file 3; Tables S9 and S10). For the analysis of the combined cohort, a subset of PZMs was created in order to ensure that the analyzed PZMs likely contribute to the phenotype (PZMs = 676) (Additional file 1; Table S11). For that purpose, individuals with germinal mutations in ASD risk genes (SFARI scores 1 and 2) were eliminated from the PZMs dataset. Thus, germinal DNMs and the subset of PZMs from the combined cohort were used in the analysis (germinal DNMs = 2270; PZMs = 676) (Additional file 3; Table S12 and S13). The analysis was also run independently in unaffected siblings using data previously published by the ASC (germinal DNMs = 780; PZMs = 239) (Additional file 1; Table S14).

The gene name alias and the gene size matrix provided by DNENRICH were used as input files used in this analysis together with the following gene-sets: (1) FMRP target genes identified by Darnell et al.^26^ and downloaded from Genebook (http://zzz.bwh.harvard.edu/genebook/) (N = 788); (2) Genes included in the GO:0006325 chromatin organization (N = 723) (http://www.geneontology.org/); (3) Synaptic genes (N = 903)^27^; (4) Human orthologs of genes essential in mice (N = 2472)^28^; (5) CHD8 target genes in human mid fetal brain (N = 2725)^29^; (6) List of SFARI genes (N = 990) (https://gene.sfari.org/autdb/HG_Home); (7) LoF intolerant genes (pLi > 0.9) (N = 3230)^29,30^; 8) RBFOX target genes (N = 587)^31^; (9) miR-137 target genes (N = 428)^32^; (10) CELF-4 target genes (N = 954)^33^; (11) Allele biased genes in differentiating neurons (N = 802)^34^; (12) Known intellectual disability genes (N = 1547)^35^; (13) Intergenic and Intronic Brain Expressed Enhancers (BEE) (N = 673)^36^; (14) Genomic intervals surrounding known telencephalon genes scanned for enhancers (N = 79)^37^; and (15) miR-138 target genes (N = 255)^38^. Empirical *p* values were obtained from one million permutations for each gene-set.

### Gene ontology enrichment analysis

An exploratory GO enrichment analysis was carried out using the Enrichr tool. This analysis allows studying if genes harboring germinal DNMs or PZMs are involved in different biological processes. To this aim, the combined dataset of germinal missense and nonsense DNMs and the subset of PZMs were employed (germinal genes = 1972; PZMs genes = 624) (Additional file 3; Table S15). In addition, the REViGO tool (http://revigo.irb.hr/) was employed to visualize GO terms in semantic similarity-based scatterplots using SimRel as a semantic similarity measure. Thus, the top 30 enriched GO terms in each group (PZM *vs* germinal) were visualized using a modification of the R script provided by the REViGO online tool.

Network visualization of the top 50 enriched terms in each group of genes was performed with Enrichment Map, a Cytoscape (v.3.6.1) plugin for functional enrichment visualization^39^. Each node represents a gene-set (GO term) and the size of the node is proportional to the number of genes participating in the GO term (overlap coefficient). Nodes were considered as connected when the overlap coefficient was greater than 0.7 and edge-width represents the overlap between gene-sets. The border-width of each node represents the corresponding *p* value for each GO term.

### Expression cell-type enrichment analysis and expression analysis across brain regions and developmental periods

Expression Weighted Cell-type Enrichment (EWCE) method (https://github.com/NathanSkene/EWCE) was used to explore whether genes harboring germinal DNMs (N = 1972) and genes harboring PZMs (N = 624) (Additional file 3; Table S15) were differentially expressed across several neuronal cell types. EWCE involves testing whether the given genes in a target list have higher levels of expression in a given cell type compared to what is expected by chance. Brain single-cell transcriptomic data from Karolinska Institute (ctd_allKI) was used for the EWCE analysis. Brain regions included in the KI mouse super dataset are the neocortex, hippocampus, hypothalamus, striatum, and midbrain, as well as samples enriched for oligodendrocytes, dopaminergic neurons and cortical parvalbumin interneurons (total cells = 9970). Background gene-set comprises all human-mice orthologous. Probability distribution for our gene lists was calculated by randomly sampling 100,000 genes from the background set controlling for transcript length and GC content. Bootstrapping function was then applied on level 1 annotation.

pSI (specificity index statistic), an R package, was employed to study the expression of genes harboring PZMs and germinal mutations across different brain regions and neurodevelopmental periods^40,41^. Lists of specifically expressed human genes (human.rda) obtained from BrainSpan data (gene-sets for 6 brain regions and gene-sets for 10 developmental periods) were employed. The Fisher iteration test included in the pSI package was used to analyze if the listed genes harboring PZMs and germinal mutations were significantly overrepresented. Brain areas significantly enriched with PZMs or germinal genes in specific developmental periods (*p* adjusted value < 0.05) were represented as a matrix. Biorender (https://biorender.com) was used to draw the brain images (Fig. 6).

### Ethics approval and consent to participate

The corresponding Research Ethics Committee of Galicia approved our study: (Comité Ético de Investigación Galicia (the only IEC authorized in this autonomous region); Number: 2012/098; Approval: 28-June-2012; Title: Contribución a la búsqueda de las causas genéticas de los trastornos del espectro autista. All participants, parents or legal representatives provided written informed consent at enrollment and the study was conducted according to the declaration of Helsinki. The ASC data employed in this study were already published (https://doi.org/10.1038/nn.4598). The corresponding ethics committee has approved these genetic data.

## Results

### Transmission and de novo association test (TADA-denovo)

Transmission and De novo Association test (TADA) assesses if a gene is affecting ASD risk based on several parameters: the gene mutation rate, the recurrence of DNMs in the gene and the severity of the mutations^22^. Thus, TADA-Denovo analysis was independently run in both datasets (germinal and PZMs genes). The main aim of TADA-Denovo is to identify those genes that could be differentially involved in ASD etiology depending on the type of DNMs harbored by them (germinal or PZMs). We focused the analysis on damaging mutations (LoF and likely pathogenic missense variants) to increase the likelihood of finding “strong” candidate genes. First, the set of genes from the Spanish cohort (360 trios) (germinal genes = 181; PZMs genes = 105) was analyzed. The analysis of the germinal gene list identified 12 genes with an FDR < 0.3 (Table 1 and Additional file 2; Table S16) including 3 genes (*SCN2A, ARID1B* and *CHD8*) with an FDR < 0.1. The analysis of the PZMs gene list identified 13 genes with an FDR < 0.3 (Table 1 and Additional file 2; Table S17) of which 4 genes (*KMT2C, FRG1, GRIN2B* and *MAP2K3*) had an FDR < 0.1.

**Table 1:** ASD risk genes carrying germinal DNMs and PZMs in the Spanish cohort.

Genes	q-value	*p* value	Mutations
*SCN2A*	0.004	2.76 × 10^–7^	Germinal
*ARID1B*	0.050	2.76 × 10^–6^	Germinal
*CHD8*	0.066	3.31 × 10^–6^	Germinal
*FIG4*	0.103	9.39 × 10^–5^	Germinal
*RBM15*	0.126	1.16 × 10^–5^	Germinal
*HUWE1*	0.148	3.54 × 10^–5^	Germinal
*KIAA1107*	0.188	5.08 × 10^–5^	Germinal
*VWAS5B1*	0.218	5.08 × 10^–5^	Germinal
*EMCN*	0.242	6.13 × 10^–5^	Germinal
*SH2B2*	0.262	7.90 × 10^–5^	Germinal
*ASMT*	0.277	9.34 × 10^–5^	Germinal
*MYLK4*	0.291	9.67 × 10^–5^	Germinal
*KMT2C*	0.001	4.76 × 10^–7^	PZM
*FRG1*	0.015	4.46 × 10^–7^	PZM
*GRIN2B*	0.040	4.76 × 10^–7^	PZM
*MAP2K3*	0.08	6.67 × 10^–6^	PZM
*SRGAP2*	0.106	7.62 × 10^–6^	PZM
*MBD6*	0.124	7.62 × 10^–6^	PZM
*POTEB2*	0.168	2.57 × 10^–5^	PZM
*CALML6*	0.200	2.67 × 10^–5^	PZM
*PRDX6*	0.226	3.05 × 10^–5^	PZM
*SSR2*	0.245	3.14 × 10^–5^	PZM
*VEGFA*	0.264	4 × 10^–5^	PZM
*CANX*	0.278	0.0001	PZM
*ZNF276*	0.290	0.00012	PZM

*p* values and q-values were obtained after running TADA-Denovo using germinal DNMs and PZMs from the Spanish ASD cohort (N = 360). Only genes with q-values < 0.3 are shown.

**Figure 1:**
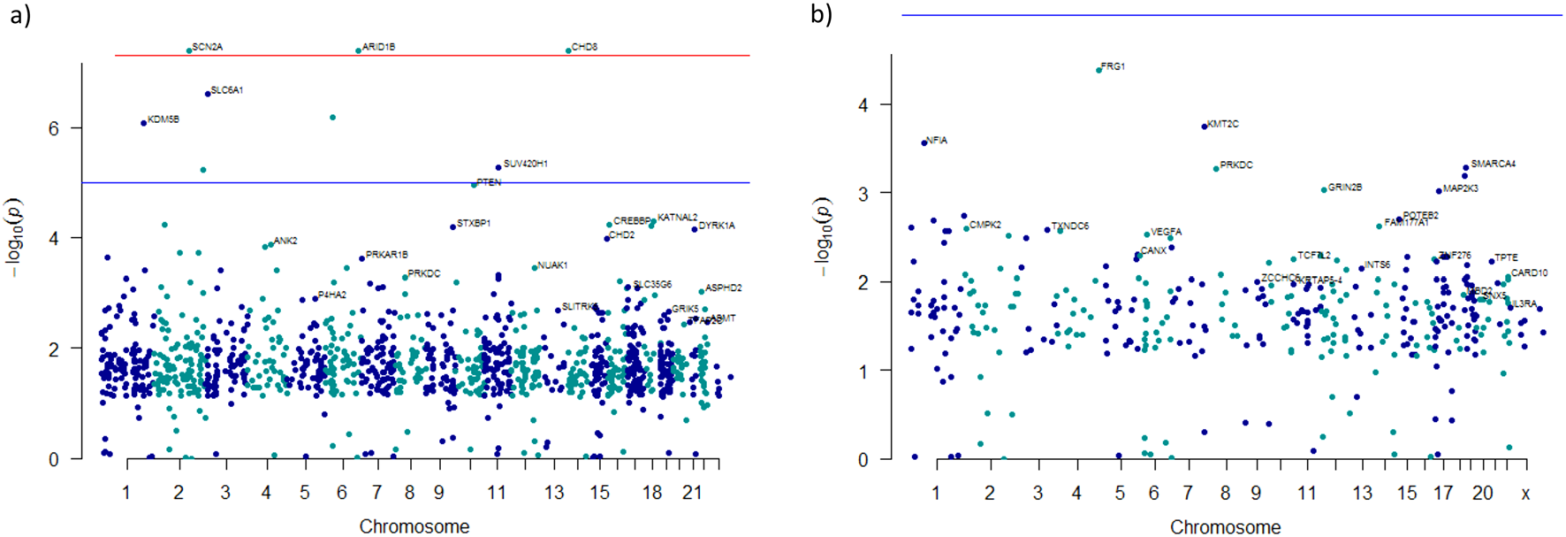
Manhattan plots depicting the ASD risk genes prioritized by TADA-Denovo (chromosome and log10 *p* value for each gene are represented in axis x and y). (**a**) *p* values were obtained from analysis of germinal mutations in the combined cohort using TADA-Denovo. Red line represents the *p* value < 1 × 10^–8^ and blue line *p* value < 1 × 10^−5^. (**b**) *p* values were obtained from analysis of PZMs in the combined cohort using TADA-Denovo. Blue line represents *p* value < 1 × 10^−5^.

In the combined cohort (genes from the Spanish cohort plus genes from the Lim et al. publication^18^ (germinal genes = 1210; PZMs genes = 362) TADA identified 34 genes with an FDR < 0.1 (Table 2 and Fig. 1a) and 103 genes with an FDR < 0.3 (Additional file 2; Table S18). Three of the genes *(SCN2A, ARID1B, CHD8*) with germinal DNMs were prioritized (FDR < 0.1) both in the combined cohort and in the Spanish cohort when TADA was employed. Analysis of PZMs genes in the combined cohort identified three genes (*FRG1, KMT2C* and *NFIA*) with an FDR < 0.1, and 14 genes with an FDR < 0.3 (Table 2 and Fig. 1b; Additional file 2; Table S19). Only two genes, *KMT2C* and *FRG1*, have remained significant after FDR correction (< 0.1) in both the Spanish and the combined cohort.

**Table 2:** ASD risk genes carrying germinal DNMs and PZMs in the combined cohort.

Gene	q-value	*p* value	Mutations
*SCN2A*	5.04 × 10^–12^	4.13 × 10^–8^	Germinal
*CHD8*	2.40 × 10^–5^	4.13 × 10^–8^	Germinal
*ARID1B*	5.36 × 10^–5^	4.13 × 10^–8^	Germinal
*SLC6A1*	0.00015	2.48 × 10^–7^	Germinal
*SYNGAP1*	0.0005	6.61 × 10^–7^	Germinal
*KDM5B*	0.0008	8.26 × 10^–7^	Germinal
*SUV420H1*	0.002	5.37 × 10^–6^	Germinal
*TRIP12*	0.003	5.79 × 10^–6^	Germinal
*PTEN*	0.004	1.14 × 10^–5^	Germinal
*KATNAL2*	0.008	5.01 × 10^–5^	Germinal
*NRXN1*	0.012	5.79 × 10^–5^	Germinal
*CREBBP*	0.02	5.92 × 10^–5^	Germinal
*CELF4*	0.02	6.09 × 10^–5^	Germinal
*STXBP1*	0.02	6.48 × 10^–5^	Germinal
*DYRK1A*	0.02	7.09 × 10^–5^	Germinal
*CHD2*	0.03	0.0001	Germinal
*ANK2*	0.03	0.0001	Germinal
*WDFY3*	0.03	0.0001	Germinal
*UNC80*	0.04	0.0002	Germinal
*CLASP1*	0.04	0.0002	Germinal
*TMEM39B*	0.05	0.0002	Germinal
*PRKAR1B*	0.05	0.0002	Germinal
*USP45*	0.05	0.0003	Germinal
*NUAK1*	0.06	0.0004	Germinal
*NAA15*	0.06	0.0004	Germinal
*FOXP1*	0.07	0.0004	Germinal
*ZC3H11A*	0.07	0.0004	Germinal
*DPP3*	0.07	0.0005	Germinal
*PRKDC*	0.08	0.0005	Germinal
*ATP1A1*	0.08	0.0005	Germinal
*LRP5*	0.09	0.0005	Germinal
*SLC12A3*	0.09	0.0006	Germinal
*FBXO18*	0.096	0.0006	Germinal
*PTK7*	0.0999	0.0007	Germinal
*FRG1*	0.04	4.14 × 10^–3^	PZM
*KMT2C*	0.07	0.00018	PZM
*NFIA*	0.09	0.00028	PZM
*SMARCA4*	0.12	0.00052	PZM
*PRKDC*	0.13	0.00055	PZM
*KLF16*	0.15	0.00064	PZM
*GRIN2B*	0.17	0.00095	PZM
*MAP2K3*	0.18	0.00098	PZM
*HNRNPU*	0.21	0.0019	PZM
*POTEB2*	0.23	0.002	PZM
*RNPC3*	0.25	0.002	PZM
*FAM177A1*	0.27	0.002	PZM
*CALML6*	0.28	0.002	PZM
*CMPK2*	0.3	0.003	PZM

*p* values and q-values were obtained after running TADA-Denovo using germinal DNMs and PZMs from the combined cohort (N = 2103). Genes with q-values < 0.1 are shown in the case of germinal DNMs and genes with q-value < 0.3 are shown in the case of PZMs.

A total of 17 genes (50%) from the set of germinal genes in the combined cohort (34 genes, FDR < 0.1) were identified as “high confidence” or “strong” ASD candidates following SFARI Gene scoring criteria (scores 1, 2, 1 s and 2 s). In addition, 11 of these genes (64, 70%) have shown an FDR < 0.1 in previous TADA analysis^6^. Moreover, 10 of the remaining genes identified by TADA (FDR < 0.1) are included in SFARI gene lists (scores 3, 4 and 5) and 5 of the genes identified by TADA were reported in relation with another disease (not ASD) by OMIM database (Additional file 4; Table S20).

PZM analysis has shown association of *KMT2C* (SFARI score s2) as well as other 3 genes FDR < 0.1). It is worth to note that *NFIA* has been previously reported as a plausible candidate gene in ASD (SFARI score 4) but this is the first time that *FRG1* is reported in ASD. *SMARCA4, PRKDC, KLF16, GRIN2B* and *HNRNPU* (SFARI score 3 and 4) were among those plausible ASD candidate genes previously identified with an FDR value between 0.1 and 0.3. GRIN2B was previously reported by SFARI as a strong ASD risk gene (score 1) (Additional file 4; Table S21).

### Gene-set enrichment analysis of PZMs and germinal mutations

DNENRICH was run to estimate a statistical significance of enrichment for germinal and PZMs within previously ASD and NDDs associated gene-sets. Synonymous mutations were excluded from the analysis because they are unlikely to contribute to ASD phenotype and only nonsense and missense mutations were considered. First, gene-set enrichment analysis was performed using the list of genes and DNMs (germinal and PZMs) from the Spanish cohort (Additional file 3; Tables S9 and S10) against several background gene lists (see “Methods” section). Our results indicate that germinal genes shown enrichment in several gene-sets (germinal genes = 228, germinal DNMs = 236): FMRP target genes (*p* value = 0.003), known intellectual disability genes (*p* value = 0.0073), LoF intolerant genes (*p* value = 0.002), SFARI genes (*p* value = 1 × 10^–7^) and genes involved in chromatin organization (*p* value = 0.00018) (Table 3). However, only the LoF intolerant gene-set has shown association with the list of PZMs genes (PZMs genes = 155, PZMs = 164) (Table 3).

**Table 3:** Results of the gene-set enrichment analysis for the list of genes harboring germinal DNMs and PZMs (Spanish cohort).

Gene-sets	*p* value	Observed mutations	Expected mutations	Analysis
SFARI genes	1 × 10^–6^	49	20.9601	Germinal
FMRP targets	0.00013	39	20.9788	Germinal
Genes involved in chromatin organization	0.00018	24	10.7377	Germinal
LoF intolerant genes	0.0018	79	58.9156	Germinal
Known ID genes	0.0073	40	27.0286	Germinal
Essential genes	0.0709	49	39.968	Germinal
CHD8 targets	0.226	39	34.4983	Germinal
mir137 targets	0.3251	8	6.49354	Germinal
Synaptic genes	0.3592	14	12.4002	Germinal
Genomic intervals surrounding known telencephalon genes scanned for enhancers	0.5386	1	0.771873	Germinal
Alelle biased genes in differentiating neurons	0.6	12	12.502	Germinal
CELF4 targets	1	0	0.05078	Germinal
mir128 targets	1	0	0.022676	Germinal
RBFOX targets	1	0	0.055409	Germinal
LoF intolerant genes	0.0283	51	39.9973	PZM
Genes involved in chromatin organization	0.0625	12	7.28793	PZM
FMRP targets	0.0764	20	14.2422	PZM
Sfari genes	0.0764	20	14.2361	PZM
CHD8 targets	0.0884	30	23.4115	PZM
Genomic intervals surrounding known telencephalon genes scanned for enhancers	0.0977	2	0.524631	PZM
Essential genes	0.234	31	27.1352	PZM
Synaptic genes	0.3339	10	8.41702	PZM
Known ID genes	0.7554	16	18.3456	PZM
mir137 targets	0.8198	3	4.40692	PZM
Alelle biased genes in differentiating neurons	0.9727	4	8.48746	PZM
CELF4 targets	1	0	0.034487	PZM
mir128 targets	1	0	0.015316	PZM
RBFOX targets	1	0	0.037786	PZM

**Figure 2:**
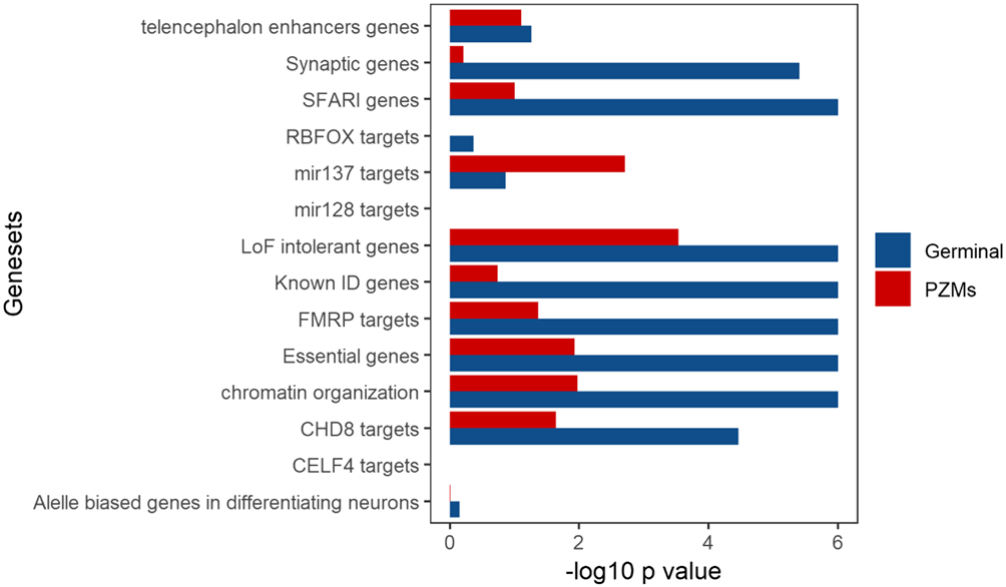
Gene-set enrichment analysis using germinal and PZM from the combined cohort. Gene-set enrichment analysis was done with DNENRICH. − log10 *p* value for each gene-set is shown for each type of mutation and tested gene-set.

DNENRICH analysis in the combined cohort demonstrated enrichment for several gene-sets for both germinal and PZMs genes: chromatin organization, SFARI genes, LoF intolerant genes, CHD8 target genes and essential genes. In addition, the germinal gene list (germinal genes = 1972, germinal DNMs = 2270) showed enrichment for FMRP target genes (*p* value = 1 × 10^–6^), known intellectual disability genes (*p* value = 1 × 10^–6^) and synaptic genes (*p* value = 4 × 10^–6^) (Table 4, Fig. 2). PZMs genes (PZMs genes = 624, PZMs = 676), have only shown association in the case of the miR-137 target gene-set (*p* value = 0.0019) (Table 4, Fig. 2). The same analysis was performed using the list of germinal genes from unaffected siblings (germinal genes = 744, germinal DNMs = 780; PZMs genes = 237, PZMs = 239) (Additional file 1; Table S14). A significant enrichment was identified only with the FMRP targets gene-set (data not shown).

**Table 4:** Results of the gene-set enrichment analysis for the list of genes harboring germinal DNMs and PZMs (combined cohort).

Gene-set	*p* value	Observed mutations	Expected mutations	Analysis
Essential genes	1 × 10^–6^	533	404.162	Germinal
FMRP targets	1 × 10^–6^	331	212.218	Germinal
Known ID genes	1 × 10^–6^	373	273.213	Germinal
LoF intolerant genes	1 × 10^–6^	733	595.59	Germinal
SFARI genes	1 × 10^–6^	434	211.973	Germinal
Synaptic genes	4 × 10^–6^	180	125.408	Germinal
Genes involved in chromatin organization	1 × 10^–6^	168	108.449	Germinal
CHD8 targets	3.5 × 10^–5^	420	348.544	Germinal
Genomic intervals surrounding known telencephalon genes scanned for enhancers	0.0550	13	782.029	Germinal
mir137 targets	0.1368	75	657.234	Germinal
RBFOX targets	0.4299	1	0.562709	Germinal
Alelle biased genes in differentiating neurons	0.6999	121	126.324	Germinal
CELF4 targets	1	0	0.51357	Germinal
mir128 targets	1	0	0.22838	Germinal
SFARI genes	1 × 10^–7^	127	612.987	PZM
LoF intolerant genes	0.0003	212	172.214	PZM
mir137 targets	0.0019	33	190.234	PZM
Genes involved in chromatin organization	0.0106	45	313.424	PZM
Essential genes	0.0118	140	116.895	PZM
CHD8 targets	0.0228	120	100.751	PZM
FMRP targets	0.0425	75	614.294	PZM
Genomic intervals surrounding known telencephalon genes scanned for enhancers	0.0796	5	22.672	PZM
Known ID genes	0.1840	87	790.127	PZM
Synaptic genes	0.6109	35	362.813	PZM
Alelle biased genes in differentiating neurons	0.9744	26	364.938	PZM
CELF4 targets	1	0	0.147914	PZM
mir128 targets	1	0	0.066195	PZM
RBFOX targets	1	0	0.162842	PZM

### Gene ontology enrichment analysis

**Figure 3:**
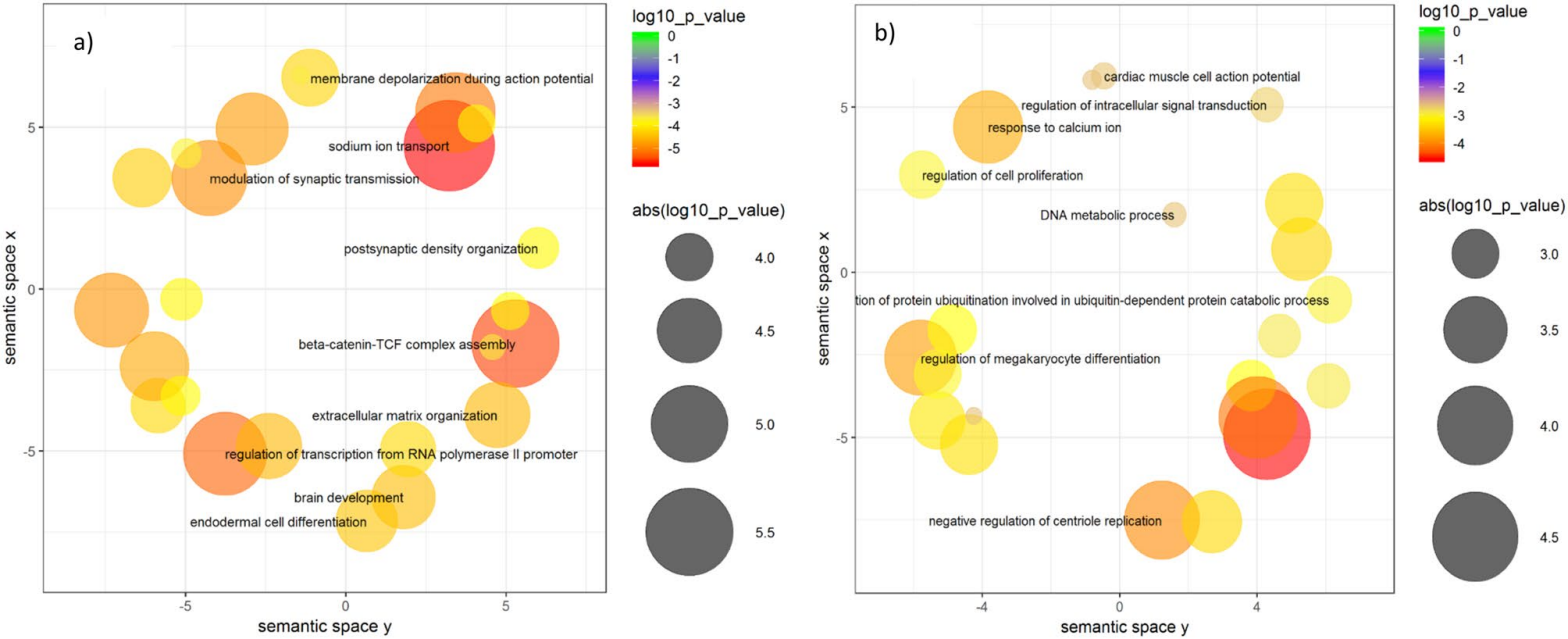
Scatterplots representing the top 30 significant biological processes (combined cohort). (**a**) Top 30 biological processes enriched in genes harboring germinal DNM are shown. (**b**) Top 30 biological processes enriched in genes harboring PZMs DNMs are shown.

GO enrichment analysis revealed remarkable differences between germinal and PZMs gene lists from the combined cohort (Additional file 3; Table S15). The germinal set showed a significant enrichment in different GO terms related to synaptic function and transcription regulation. In particular, it is worth to note the association of GO terms related to ion transport: GO:0006814, Benjamini-Hochberg-corrected p[Pbh] = 0.005; GO0035725, Pbh = 0.005 and GO0006816, Pbh = 0.006 (Additional file 4; Table S22, Fig. 3a). The GO terms enriched in the subset of PZMs are related to regulation of gene expression, biosynthesis, differentiation or migration: GO0010629, p[Pbh] = 0.074; GO:2000113, p[Pbh] = 0.092; GO:0045652, p[Pbh] = 0.092; GO:0030336, p[Pbh] = 0.0995. (Additional file 4; Table S23, Fig. 3b).

**Figure 4:**
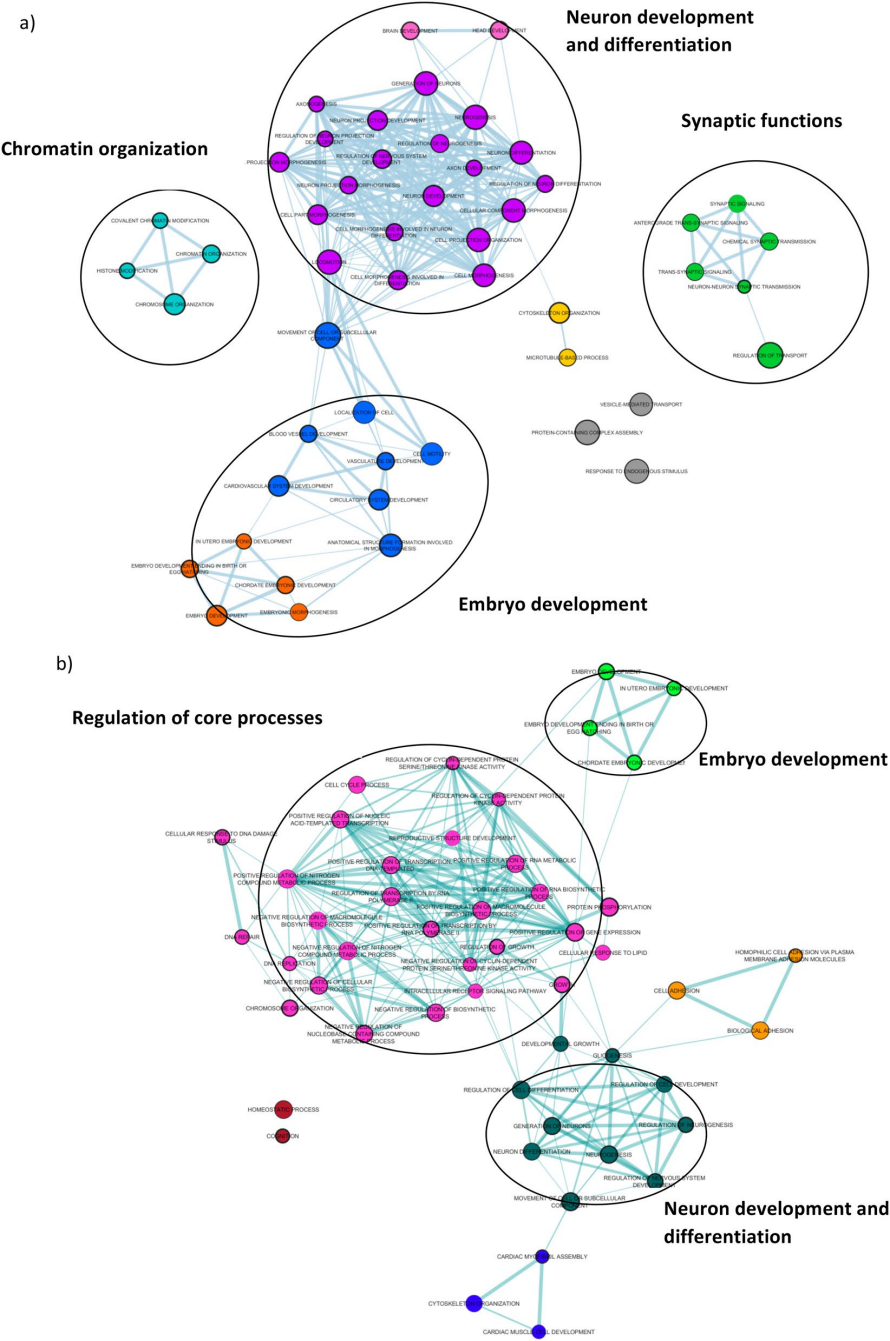
Visualization of the top 50 GO terms grouped by clusters of biological functions (DNMs obtained from the combined cohort) (**a**) GO terms clusters for genes harboring germinal mutations. (**b**) GO terms clusters for genes harboring PZMs.

GO enrichment analysis was depicted by semantically clustering the top 50 enriched terms for germinal and PZMs gene lists. The germinal gene list resulted in three differentiated clusters: neuron development and differentiation, synaptic functions, and chromatin modifications. The existence of a fourth cluster, which includes terms related to embryonic development, was also highlighted (Fig. 4a). In the case of PZMs genes, all the clusters were partially related to each other. However, we identified another cluster that includes terms related to the regulation of core processes (e.g., protein phosphorylation, regulation of growth, negative regulation of cellular biosynthetic processes, positive regulation of transcription DNA template). It is also important to highlight the GO terms related to neuron and embryonic development (Fig. 4b).

### Expression cell-type enrichment analysis and expression analysis across brain regions and developmental periods.

**Figure 5:**
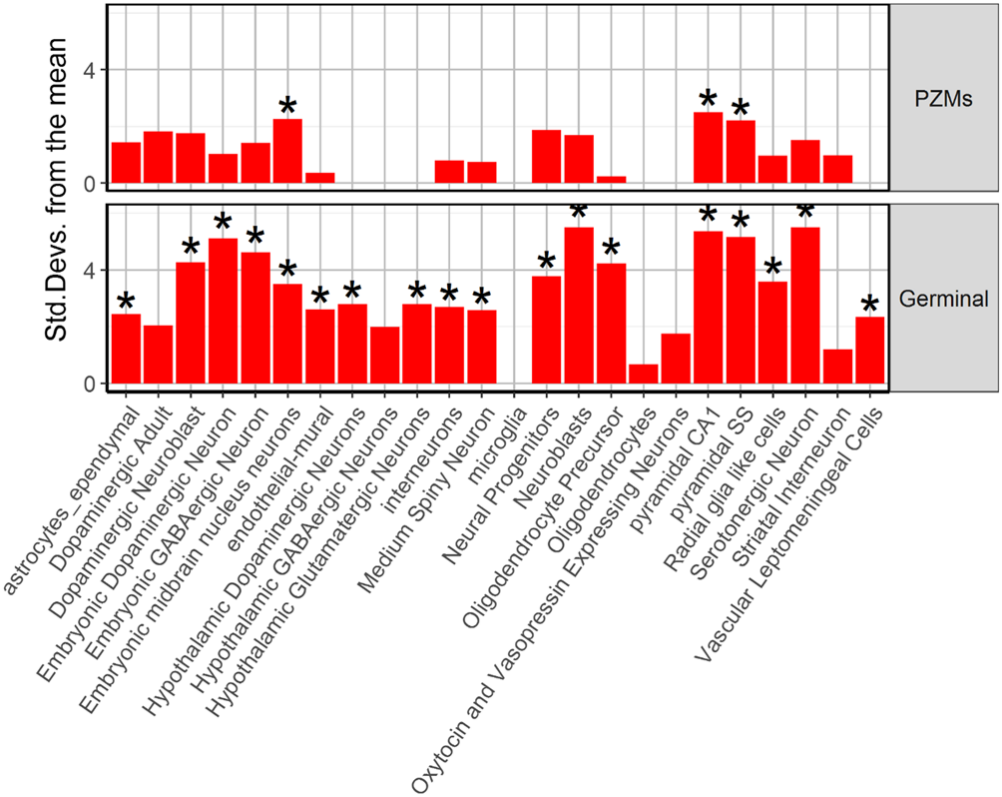
Expression Weighted Cell type Enrichment for PZM and germinal genes.

First, we examined whether germinal genes or PZMs genes from the combined cohort, were differentially expressed in the transcriptome dataset corresponding to level 1 cell types. As expected, germinal genes were significantly enriched in several cell types (Additional file 3; Table S24; Fig. 5). The most enriched cell types were those related to neurotransmission (dopaminergic neuroblast; *p* value < 0.0001; embryonic dopaminergic neurons, *p* value < 0.0001; embryonic GABAergic neurons, *p* value < 0.0001; serotonergic neurons, *p* value < 0.0001). The PZMs gene list showed enrichment for three different cell types: pyramidal CA1 neurons; *p* value = 0.0066, pyramidal somatosensory (SS); *p* value = 0.0152, embryonic midbrain nucleus neurons; *p* value = 0.0185 (Additional file 3; Table S25, Fig. 5).

**Figure 6:**
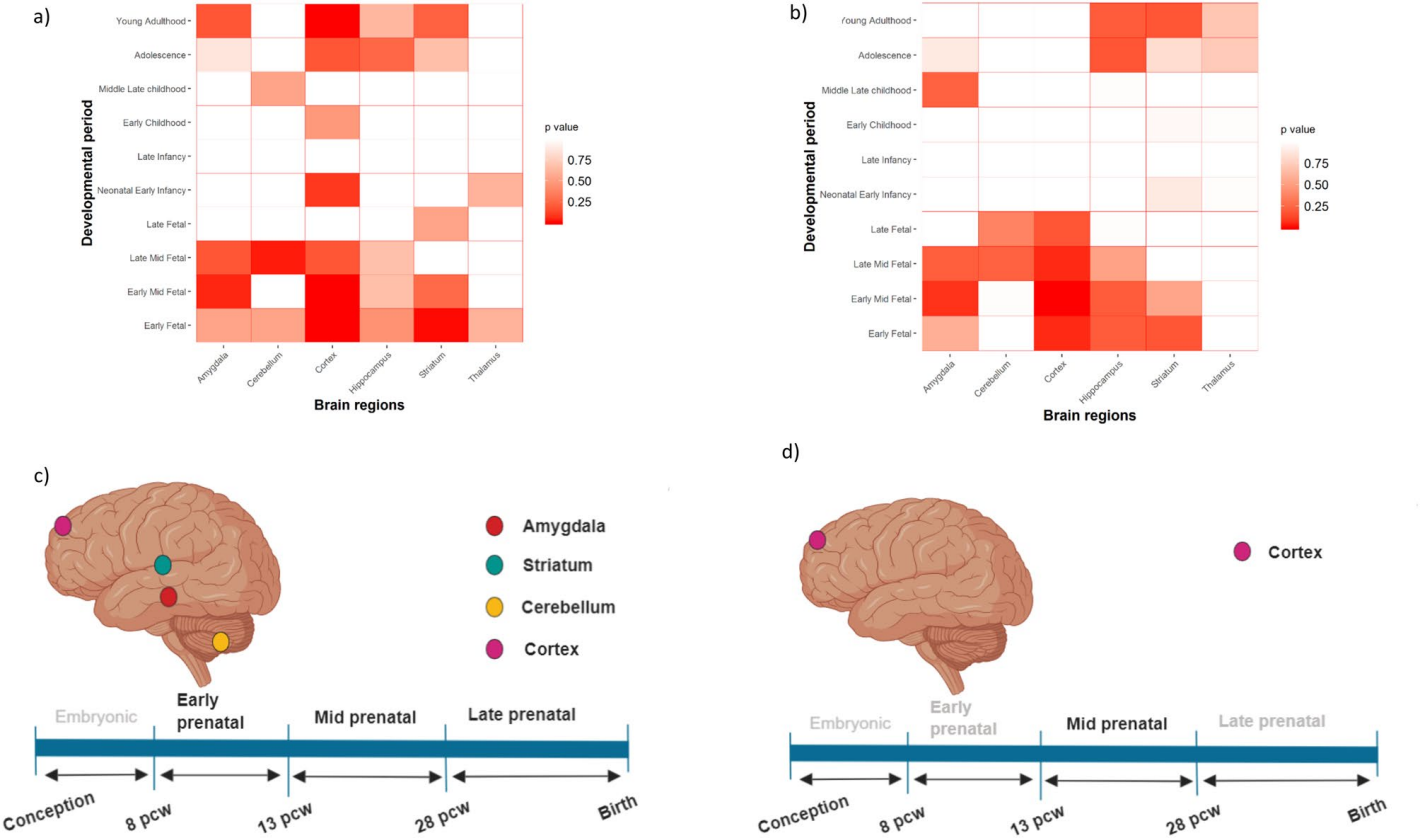
Expression analysis across developmental stages and brain areas for germinal and PZMs genes from the combined cohort. Expression gene sets were obtained from BrainSpan. (**a**,**c**) Expression of germinal genes across different brain regions and developmental periods. (**b**,**d**) Expression of PZMs genes across different brain regions and developmental periods.

To gain insight into the spatiotemporal distribution, we analyzed the expression of germinal and PZMs genes (Additional file 3; Table S15) across several brain regions and different neurodevelopmental periods obtained from BrainSpan. Germinal genes were significantly expressed in the cortex, striatum, cerebellum and amygdala in prenatal stages (early, early mid and late) (Additional file 3; Table S26, Fig. 6a,c). PZMs genes were significantly expressed in the cortex during the early mid-fetal period. Although we did not find found a significant enrichment in other brain areas or neurodevelopmental periods for PZMs, *p* values close to the significance threshold were found in cortex (early, late mid-fetal) and amygdala (late mid-fetal) (Additional file 3; Table S27, Fig. 6b,d).

## Discussion

ASD is a complex NDD, characterized by its clinical and genetic heterogeneity. It is estimated that over 1000 genes are involved in causing ASD but it is difficult to track them down and to functionally characterize them. Thus, it is widely believed that the vast majority of ASD genetic factors remain largely unknown^6^. In addition, DNMs are a strong source of genetic causality in ASD even though they have been usually identified in a germinal state. This means that most of the ASD genetic studies do not consider DNMs that appear post-zygotically. Recent studies have suggested that some ASD candidate genes could carry more mosaic mutations than others^15–18^. However, the phenotypic effect of PZMs and its impact in clinical diagnosis is not completely understood. A recent study showed that brain malformations can occur when around 10% of peripheral blood cells harbor the mosaic mutation^42^. In addition, mosaic mutations could act as phenotype modifiers, resulting in patients who have less severe symptoms^43^.

This study has explored the possibility of finding new genetic risk factors in ASD when PZMs are also considered as well as it was focused on the deepening of their biological role. Three genes were associated in the PZM analysis done by TADA (q-value < 0.1; *FRG1*, *KMT2C* and *NFIA*) in the combined cohort. *FRG1* has not been previously associated with NDDs while both, *KMT2C* and *NFIA,* have been previously reported as possible implicated in ASD and intellectual disability^44,45^. However, none of these genes were associated to ASD in the germinal analysis, which seems to point that they tend to differentially harbor PZMs.

Facioscapulohumeral muscular dystrophy (FSHD) region gene 1 (*FRG1*) (also other aliases) encodes a cytoplasmic protein involved in muscular and vascular development. *FRG1* mapped 100 kb centromeric of the repeated units on chromosome 4q35 that contribute to the development of Facioscapulohumeral Muscular Dystrophy (FSHD, OMIM #158900)^46^. Although the molecular pathogenesis of FSHD remains unveiled, it is a muscular disease that shows comorbidity with several neurological symptoms such as epilepsy or intellectual disability^47^. *FRG1* can act as a splicing regulator and regulates the expression of *Rbfox1,* an RNA-binding protein that it is involved in neuronal migration and synapse formation*.* Mutations in *RBFOX1* have been associated with ASD^48,49^*.* Our study reveals the association of *FRG1* in ASD when PZMs are considered. *FRG1* association was not reported in the previous Lim et al. publication because it can be explained by novel nonsense PZM found in the Spanish cohort (Additional File 1, Table S1). However, this association should be interpreted with caution because *FRG1* is not constraint for truncating (pLI = 0) or for missense variants (Z = 0.18).

*KMT2C (lysine (K)-specific methyltransferase 2C)* encodes a methyltransferase that regulates gene transcription. De novo LoF mutations in *KMT2C* have been detected in individuals with Kleefstra syndrome 2 (OMIM #617768). Kleefstra syndrome is caused by haploinsufficiency of the euchromatin histone methyltransferase 1 (*EHMT1*) and characterized by ASD and other comorbid symptomatology as intellectual disability and a delayed psychomotor development. These symptoms could be explained by a molecular interplay between *KMT2C* and *EHMT1* both involved in the regulation of synaptic plasticity in the adult brain^44^. WES focused on the detection of PZMs using new bioinformatic pipelines have found mosaic mutations within *KMT2C*^15^. These previous findings and the association of *KMT2C* in our study seem to point that some genes previously identified as high-confidence ASD risk genes can also harbor PZMs.

The third gene associated in the PZM analysis is *NFIA (Nuclear Factor I A).* It encodes a member of the NF1 (nuclear factor 1) family of transcription factors determining the regulation of gliogenesis and other neuronal processes^50^. Although two DNMs in *NFIA* have been previously reported in patients with intellectual disability, there is no reliable evidence to consider this gene as a high-confidence ASD risk gene^51^. However, it is worth noting that an *NFIA*-related disorder caused by inherited or DNMs has been reported. This syndrome is characterized by brain malformations and it is usually accompanied by renal urinary tract defects (OMIM #613735)^52^. The clinical presentation of the syndrome is highly variable and rarely all the described features are present in one individual^53^. To our knowledge, this is the first time that *NFIA* is pointed as a plausible candidate gene in ASD. This association would have been dismissed without the analysis of PZMs.

The fact that different genes across the genome carry different types of DNMs (germinal and/or PZM), could be due to the fact that some mutations can be lethal in a germinal state but not in a mosaic state. This is the case, for example, of Rett syndrome, in which *MECP*2 mutations are lethal in males and dominant in females, but in a few cases, mosaic mutations have been reported to be compatible with male viability^54^. However, our results, interpreted in conjunction with previous genetic and phenotypic studies, seem to point to other reasonable hypothesis: clinical manifestations caused by PZMs are usually less severe than those due to germinal mutations. For instance, Kleefstra syndrome is caused by germinal LoF DNMs in *KMT2C* but, neverthless, PZMs cause a milder phenotype presentation^55^. Thus, patients with less severe phenotypes and core ASD features are overrepresented in our cohort, allowing the detection of *KMT2C* association in the PZM analysis done with TADA, while patients carrying germinal DNMs in the same gene might have been excluded for presenting a syndromic form. For the same reason, *NFIA* could appear associated with ASD only when the mutations are mosaic.

From the biological point of view, the gene-set enrichment analysis done with both lists of genes harboring PZMs and germinal mutations both in the Spanish and in the combined cohort showed a significant enrichment for several gene-sets previously involved in ASD pathogenesis: FMRP targets, genes involved in chromatin organization and high-confidence genes from SFARI^6^. Curiously, only the gene-set enrichment analysis for genes harboring PZMs (combined cohort) showed a significant enrichment for miR-137 targets. The fact that this association was found in the combined cohort and not in the Spanish cohort could be mainly due to the inclusion of a greater number of PZMs and a subsequent increase of the statistical power. miR-137 is a non-coding RNA with a critical role during brain development^56^. The expression of miR-137 is crucial to maintain the processes of neuronal differentiation and proliferation so it is involved in neuronal maturation, dendrite development and synaptogenesis^57–59^. In addition, common genetic variants within the gene that encodes miR-137 have been associated with ASD and schizophrenia^60,61^. In fact, the complete loss of miR-137 in mice is lethal, but a partial loss results in a phenotype that reproduces some of the core symptoms of ASD, such as repetitive behavior and impaired social functions^62^.

The GO enrichment analysis also points out to different biological functions for those genes harboring germinal DNMs and PZMs^18,63^_._Thus, GO terms mainly related to ion transport and modulation of the synaptic function were highlighted in germinal genes. However, PZMs genes were enriched in GO terms mainly related to the negative regulation of gene expression. The involvement of PZMs in gene regulation processes and not in fundamental biological processes such as synaptic transmission also supports the hypothesis that PZMs can cause milder clinical manifestations.

In order to provide a novel insight of the biological role of germinal DNMs versus PZMs in other biological hierarchies, two types of analysis have been carried out: a cell-type enrichment analysis and an spatio-temporal expression analysis across neurodevelopmental periods and brain areas. Germinal genes have shown a significant enrichment in both excitatory and inhibitory neurons while PZMs genes were mainly expressed in pyramidal neurons. In addition, it is worth to note that genes with germinal DNMs were expressed in both fetal and adult neurons across several brain areas (cortex, striatum, cerebellum and amygdala). However, the expression of genes carrying PZMs was restricted to the mid-fetal cortex. Interestingly, the study performed by Lim et al. highlighted the amygdala as the main brain area in which PZMs were enriched for expression. It should be considered that Lims study only includes PZMs within critical exons, in comparison with the current study that includes all genes carrying non-synonymous PZMs^18^ .

An overall view of our results suggests the existence of distinct biological mechanisms caused by PZMs and germinal genes that could influence ASD susceptibility in different manners. Thus, impaired neuronal communication linked to mutations in germinal genes support the theory that excitatory/inhibitory imbalance contributes to ASD^21,64^. The fact that several brain areas are similarly affected during development might explain the clinical heterogeneity in ASD and the high comorbidity with other disorders such as epilepsy or intellectual disability in the presence of germinal variants. By contrast, although a replication in larger cohorts is needed, it has been suggested that individuals harboring PZMs might be less affected in terms of cognitive abilities. In these patients, some biological processes such as neurogenesis, neuronal migration and differentiation that occur within early developmental stages might be disrupted. However, this would only take place in some brain cells while the remaining cells will maintain a normal functioning. In agreement with this hypothesis, it was found that brains of children with ASD have shown patches of abnormal laminar organization that could be the result of an altered migration of a small fraction of cells^65^.

Our results point out to a critical neurodevelopmental period during mid-fetal development in which the disruption of neurodevelopmental processes will take place. We demonstrated that ASD associated genes were expressed more often between mid-to-late fetal periods. In addition, although some genes harbor mutations in a mosaic state it is possible that the disruption of certain genes expressed during this crucial period would be enough to the later manifestation of ASD core symptoms.

## Conclusions

In conclusion, this study provides an additional insight into the role of PZMs in ASD etiology. It supports previous evidence of a pathogenic role for PZMs and their contribution to ASD risk. Moreover, our results suggest that PZMs could be involved in different biological processes and neurodevelopmental stages that germinal mutations. It has been revealed that ASD risk genes can differentially harbor germinal and PZMs, which highlights the clinical importance of the detection of both types of mutations. In addition, the integration of the genetic information (germinal and PZM) together with the phenotypical characterization could be also helpful in clinical practice.

## Supplementary information

Supplementary Information 1.

Supplementary Information 2.

Supplementary Information 3.

Supplementary Information 4.

## Data Availability

All data generated during this study are included in this published article and its supplementary information files.

## Abbreviations

AAF: Alternate allele frequency
ADI-R: Autism diagnostic interview-revised
ADOS: Autism diagnostic observation schedule
ASD: Autism spectrum disorder
BEE: Brain expressed enhancers
BF: Bayesian factor
DNMs: De novo mutations
EWCE: Expression weighted cell type enrichment
ExAC: Exome aggregation consortium
FDR: False discovery rate
FSHD: Facioscapulohumeral muscular dystrophy
GO: Gene ontology
GQ: Genotype quality
LoF: Loss of function
NDD: Neurodevelopmental disorder
OMIM: Online mendelian inheritance in man
pSI: Specifity index statistic
PZMs: Postzygotic mutations
TADA: Transmission and de novo association test
VCF: Variant call format
WES: Whole exome sequencing
